# 101 years ago: Hermann Muller's remarkable insight

**DOI:** 10.1093/genetics/iyad015

**Published:** 2023-02-27

**Authors:** James E Haber

**Affiliations:** Rosenstiel Basic Sciences Medical Research Center and Department of Biology, Brandeis University, Waltham, MA 02454-9110, USA

**Keywords:** Hermann Muller, gene, heredity, mutation, DNA, bacteriophage

## Abstract

More than 20 years before DNA was identified as the hereditary material, the Drosophila geneticist, Hermann Muller, envisioned the fundamental principles that such a molecule must have: to be auto-assembling and to be mutable but then again stable. He followed his prescient review of these properties with a remarkable prediction: learning about the hereditary material and its properties would not come from studying Drosophila, but from studying bacteria and their bacteriophages.

One of the most frustrating aspects of teaching molecular genetics is deciding what not to teach. Twenty-five years ago, my course included a number of “had-to-teach” experiments that established the fundamental concepts about the nature of DNA and of gene regulation: Luria and Delbruck's fluctuation test (Luria and Delbruck 1943); Hershey and Chase's demonstration of the agency of DNA during phage infection (Hershey and Chase 1952); Meselson and Stahl's demonstration of semi-conservative DNA synthesis (Meselson and Stahl 1958); the Pa-Ja-Mo experiment suggesting the role of a repressor in the Lac operon (Pardee *et al.* 1959) and the development of the idea of a messenger RNA (Jacob and Monod 1961); Benzer's mapping of the rII locus (Benzer 1959) and its use in the brilliant deciphering of the triplet nature of the genetic code (Crick *et al.* 1961); the demonstration of mRNA (Brenner *et al.* 1961); proof of the antiparallel strand structure of DNA by nearest neighbor analysis (Josse *et al.* 1961); phage lambda and the genetic switch (Ptashne 1967) … I could go on. But as time passed and we were overwhelmed by the astonishing advances in molecular genetics (PCR; DNA sequencing; fluorescent tagging of proteins; next-gen DNA sequencing; single cell RNA profiling; proteomics; CRISPR; and more), there was no longer time to delve into these foundational papers. Out they went.

But overlooked—even in the “old days”—is one of the most extraordinary examples of pure genetic thinking: Hermann Muller's (1922) speculations on the nature of the hereditary material. Muller's *American Naturalist* paper: “Variation due to change in the individual gene” (Muller 1922) was published a half-decade before Frederick Griffith's first demonstration of bacterial transformation and 22 years before the convincing demonstration that genes were indeed encoded in DNA (Avery *et al.* 1944).

Hermann Muller was one of the most creative and influential geneticists of the first third of the 20th century. He was one of the founding members of Thomas Hunt Morgan's “Fly Lab” at Columbia University, which included other such luminaries as Alfred Sturtevant and Calvin Bridges (Fig. 1). Their collective work established the basics of the chromosome theory of inheritance: that genes were situated on specific chromosomes; that chromosomes segregated regularly in meiosis, that crossing-over could occur between genes; that there were variants (mutations, alleles) of different genes, and much more. In work that Muller initiated in the mid 1920s, he demonstrated that X-rays could cause both mutations and chromosome rearrangements, work that would win him the 1946 Nobel Prize in Physiology or Medicine. (Two rather different accounts of Muller's role in these early days can be found in Alfred Sturtevant's *A History of Genetics* (Sturtevant 2001) and James Schwartz's *In Pursuit of the Gene* (Schwartz 2010)).

**Fig. 1.**
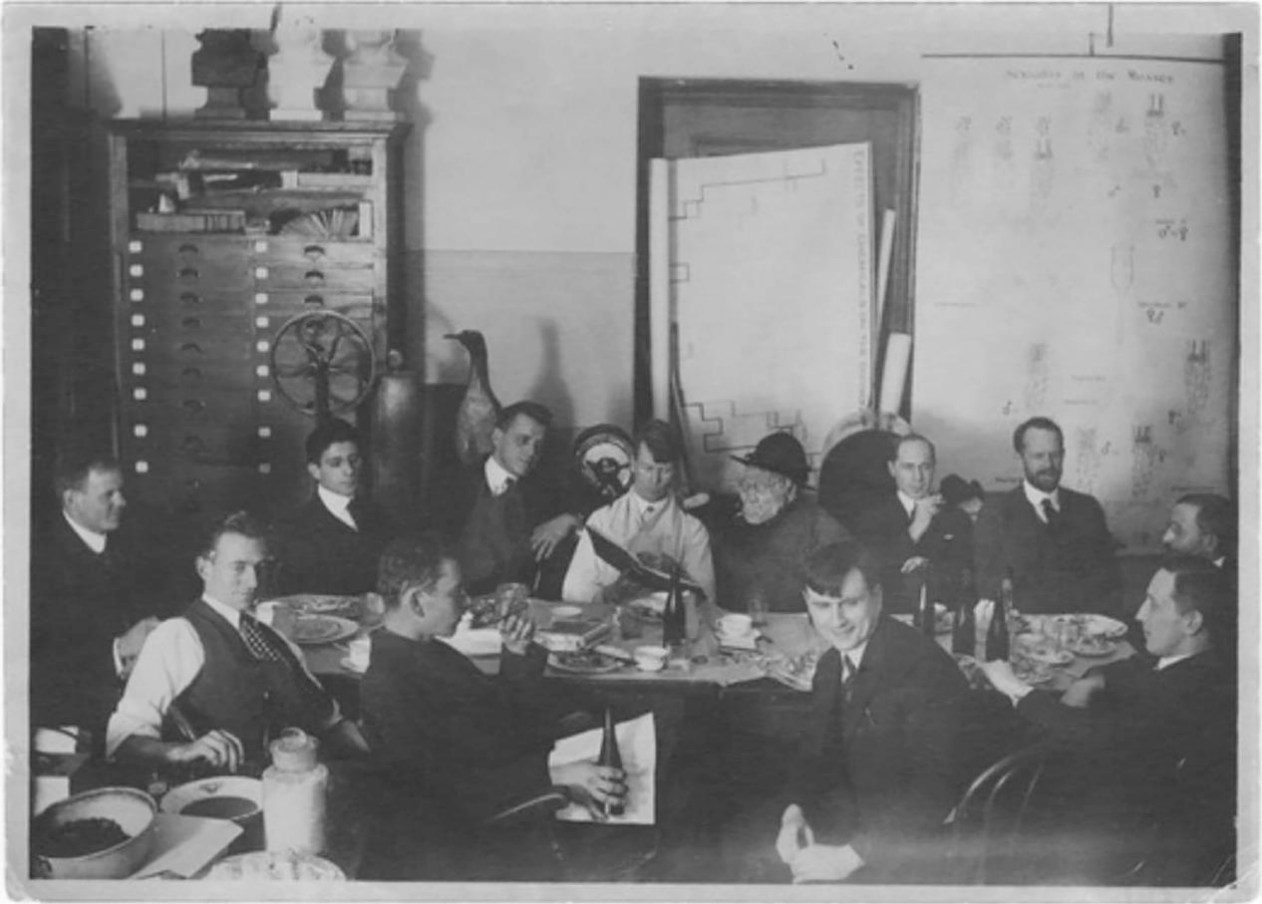
Columbia's famous Fly Room taken at a luncheon honoring Alfred Sturtevant in 1918. Muller is in the back right, sitting next to a dummy “Pithecanthropus” made for the event. To Muller's right is Thomas Hunt Morgan. Continuing clockwise, it is Frank E. Lutz, Otto L. Mohr, Alfred F. Huettner, Alfred H. Sturtevant, Franz Schrader, Ernest G. Anderson, Alexander Weinstein, S. C. Dellinger, and Calvin B. Bridges. Courtesy Lilly Library, Indiana University, Bloomington, Indiana. https://collections.libraries.Indiana.edu/muller/exhibits/show/fly-room/page-1.

Muller's (1922) essay begins with some observations that were shared by his contemporaries, based primarily on work in *Drosophila*. Genes must be ultramicroscopic bodies, given that there were hundreds of genes along a chromosome only a few microns long. In at least some cases, such as pigment production, genes “will determine the existence of a particular enzyme,” but there was no reason to suppose, as some others had, that genes were themselves enzymes.

But the key property of genes is their ability to “self-propagate,” and that “within the complicated environment of the cell protoplasm, it reacts in such a way as to convert some of the common surrounding material into an end product identical in kind with the original gene itself.” It is, he wrote, “autocatalytic” and displays “autoattraction.” Moreover, “this reaction is in each instance a rather highly localized one, since the new material is laid down by the side of the original gene.” Muller apparently reaches this conclusion from the fact that genes are arranged in a linear fashion on a chromosome and that the chromosomes of offspring retain the same gene order. More than 30 years before Watson and Crick (1953), it had not escaped Muller's attention that the original chromosome could be used as the template to produce a second.

The second remarkable property of genes is that they are mutable; but having mutated, they are again stable and heritable: “The most remarkable feature of the situation is not this oft-noted autocatalytic action in itself—it is the fact that, when the structure of the gene becomes changed, through some ‘chance variation,’ the catalytic property of the gene may become correspondingly changed, in such a way as to leave it still autocatalytic.”

“What sort of structure must the gene possess to permit it to mutate in this way? Since, through change after change in the gene, this same phenomenon persists, it is evident that it must depend upon some general feature of gene construction—common to all genes—which gives each one a general autocatalytic power—a ‘carte blanche’—to build material of whatever specific sort it itself happens to be composed of.” In this regard Muller notes that the key element in evolution is “not inheritance and variation which bring about evolution, but the *inheritance of variation*, and this in turn is due to “the persistence of autocatalysis despite the alteration in structure of the gene itself.”

But what is the physical basis of such autocatalytic, self-propagating behavior of genes? Muller considers the properties of chromosomes themselves, as seen their behavior as observed microscopically and inferred genetically. In particular “the synaptic attraction between chromosomes may be especially enlightening… because the most remarkable thing we know about genes—besides their mutable autocatalytic power—is the highly specific attraction which like genes (or local products formed by them) show for each other.” Here Muller was exceptionally lucky, because the somatic pairing of homologous chromosomes along their entire length—readily seen in *Drosophila*—is not a general feature of eukaryotes.

The forces that compel such an alignment, reflect “electromagnetic fields of force of specific patterns. To find the principle peculiar to the construction of the force-field pattern of genes would accordingly be requisite for solving the problem of their tremendous autoattraction.” Here, Muller borrows from a 1917 essay by the Harvard polymath, Leonard Troland, who argued that the autoattraction of chromosomes might be analogized to the growth of crystals, involving “similarly shaped magnets” that might attract each other. If “autocatalysis is an expression of specific attractions between portions of the gene and similar protoplasmic building blocks …, it is evident that the very same forces which cause the genes to grow should also cause the genes to attract each other, but much more strongly, since here all the individual attractive forces of the different parts of the gene are summated.” If we translate these ideas into our modern understanding of DNA, we might say that the individual base-pairings that ensure accurate copying of each base during replication will, if DNA is denatured or when a strand of DNA engages in homologous recombination, involve the “summated” attraction of many consecutive base pairs.

But then, Muller worries about the arrangement of the autoattractive forces in chromosomes. If “the parts of a molecule are in any kind of ‘solid,’ three dimensional formation, it would seem that those in the middle would scarcely have opportunity to exert the molding effect above mentioned. It therefore appears that a special manner of construction must be necessary, in order that a complicated structure like a gene may exert such an effect.” From our century-later vantage point we can re-state this idea that the structure of the gene should make it possible to use all the information available, e.g. in pairing between chromosomes; that is, there is a linear arrangement of bases in DNA in which none is irretrievably buried, though they may be wrapped and folded around histones.

I will pass over several pages where Muller offers his reflections about the origin of mutations and how they their isolation might be enhanced. He notes that J.W. Mayor had recently found an effect of X-rays on chromosome inheritance, apparently the finding that re-directed Muller's own efforts for the next many years to study the effect of X-rays on mutations and chromosome structure.

But then… as he reaches the end of his analysis, Muller offers a stunning prediction. In essence he says that if you want to really understand the structure of the gene, it will not be done with *Drosophila*. Instead, he recommends the study of “d’Herelle bodies.” In 1917, the French physician Félix d’Hérelle discovered bacteriophage. Adding a drop of an infected bacterial culture into a new culture would cause lysis of the culture. Moreover, “when a drop of the affected colony was applied to a second living colony, the second colony would be killed; a drop from the second would kill a third colony, and so on indefinitely. In other words, the substance, when applied to colonies of bacteria, became multiplied or increased, and could be so increased indefinitely; it was self-propagable. It fulfills, then, the definition of an autocatalytic substance. … Although it may really be of very different composition and work by a totally different mechanism from the genes in the chromosomes, *it also fulfills our definition of a gene*.”

Muller continues: “But the resemblance goes further—it has been found … that the substance may, through appropriate treatments on other bacteria, become changed (so as to produce a somewhat different effect than before, and attack different bacteria) and still retain its self-propagable nature.” In other words, there were mutations of the phage that altered what we would call its host range, and these mutants were again self-replicating.

And then the astonishing finale: “If these d’Hérelle bodies were really genes, fundamentally like our chromosome genes, they would give us an utterly new angle from which to attack the gene problem. They are filterable, to some extent isoluble, can be handled in test tubes, and their properties, as shown by their effects on the bacteria, can then be studied after treatment. *It would be very rash to call these bodies genes, and yet at present we must confess that there is no distinction known between the genes and them. Hence we cannot categorically deny that perhaps we may be able to grind genes in a mortar and cook them in a beaker after all. Must we geneticists become bacteriologists, physiological chemists and physicists, simultaneously with being zoologists and botanists?"*

How amazing! 1922.

Indeed, it was bacteriophage such as T2 and T4 and their *Escherichia coli* host that became the object of intense study by bacteriologists and physicists-turned-biologists in the 1940s, 1950s and 1960s, once the chemical nature of DNA had been established. These efforts yielded many of the early fundamental discoveries in molecular biology that are found in the papers I no longer have room to include in my course.

Some of the phenomena that Muller invoked remain poorly understood. In flies, homologous chromosomes pair, but how? Underneath it all must be the DNA sequence, but there is little evidence that the “auto-attraction” actually involves direct DNA base-pairing. Whether there are proteins bound to specific sequences or that long noncoding RNAs play some role remains an active area of investigation. And why don’t mammalian chromosomes pair up, although X chromosomes “kiss” during the process of X-inactivation.

The British author, David Lodge, created a character in his novel, *Small World*, who wrote his thesis on the influence of T.S. Eliot on Shakespeare (Lodge 1984). This is not a silly idea and we have the same problem in looking back 101 years on Muller's essay, inserting concepts that were completely unknown to him. And yet, he really had a vision, one that is worth reading even now.
